# Time Matters Differently in Leisure Experience for Men and Women: Leisure Dedication and Time Perspective

**DOI:** 10.3390/ijerph16142513

**Published:** 2019-07-14

**Authors:** Nuria Codina, José V. Pestana

**Affiliations:** Department of Social Psychology and Quantitative Psychology, University of Barcelona, 08035 Barcelona, Spain

**Keywords:** leisure, leisure time, leisure experience, time perspective, time budget, gender

## Abstract

There are inequalities with respect to the amount of time men and women spend on leisure. Therefore, it can be assumed that these inequalities are also manifested in the experiences derived from leisure activities and in certain attitudes to life associated with the amount of time devoted to leisure, which emphasize time orientations towards the past, present and future. Based on these ideas, this study analyses the time spent on leisure activities, leisure experience (i.e., perceptions of freedom and satisfaction), and the five factors of the time perspective (hedonistic and fatalistic present; positive and negative past; and future orientation). Participants were 435 men and 434 women, ranging from 18 to 24 years (sample mean *M* = 21.14, standard deviation *SD* = 1.99). Two tools were used: a questionnaire about leisure experience, based on the time budget technique, and the Zimbardo Time Perspective Inventory. The results show significant gender differences: men have more leisure time, but women have a more positive leisure experience and time perspectives than men. It can be concluded that women enjoy themselves more with less available leisure time and are more positive with regard to time orientations.

## 1. Introduction

In leisure research the time available is a fundamental variable [1] and the studies that compare the time men and women invest in leisure attract particular attention. Over and over again, it has been shown that there are inequalities, in different contexts and throughout life, with respect to the amount of time devoted to both leisure in general and specific leisure activities or leisure behaviour, it being women who have less available time [2,3,4,5,6,7,8,9].

These aforesaid inequalities go beyond the time devoted to leisure, because certain experiences and implications are derived from leisure [5,10,11]. In this sense, having or not having enough leisure time is associated with certain attitudes to life that lay the emphasis on the past, present or future and influence almost all aspects of human behaviour [12,13,14,15].

These appreciations put the spotlight on a new spectrum of effects and benefits associated with leisure. However, these appreciations are still not supported by a specific and sound knowledge of the particularities of this relationship. Solid research is required to defend or reject hypotheses about, on the one hand, whether gender differences are also observed in leisure experiences and in time orientations, and, on the other hand, about whether the time dedicated to leisure is associated with the aforesaid experiences and orientations. With the aim of assessing and testing these hypotheses, we consider the concepts of leisure experience, time perspective—to refer to time orientations, and previous findings linked to the joint study of both variables.

Leisure experience refers to the perceived significance of the activity for the participant, a construct with a theoretical tradition and practical application. The diverse approaches taken to the examination of its components have responded differently to the question “What is a good leisure experience?” [16], a question that represents a milestone in leisure research. As a counterpoint to the mere description of the leisure activities practiced with the corresponding details about time investments, the leisure experience makes it possible to visualize situations in which a leisure activity of short duration (for example, participating in a yoga session or attending a concert) can be experienced more positively than another activity that requires a large investment of time and resources (a three-day excursion, for example).

Among the main components of leisure discussed in the specialised literature [17,18,19,20,21,22], two essential components of the perceived leisure experience are freedom of choice and intrinsic satisfaction. As a characteristic inextricably tied to leisure [23,24], freedom of choice is understood as the perception of being able to participate in a given leisure activity without constraints, while intrinsic satisfaction is conceived as anything rewarding in itself, intrinsically motivated in general, and producing feelings of enjoyment [25]. In this sense, satisfaction is also conceived as the energetic component of motivation stimulated by the emergence of needs [21,26]. This includes interest value, which gets the person involved in an activity, and enjoyment or satisfaction, which is the assessment made of the activity carried out, prompting the person to stay involved or not [16,27,28,29,30,31]. When studying the impact of leisure activities, high values of freedom of choice related to the activity allow an examination of the so-called positive leisure experience [18]. With regard to the measurement of the leisure experience, the adaptation to the psychological approach of the time budget technique (hereinafter, TB) [30,32] is what has made it possible to describe the relationships between a leisure activity (with its respective time investment) and the perceptions derived from its practice (examples of this technique in [33,34,35,36,37,38].

Time perspective (hereinafter, TP) is a process situated at the core of individual and social behaviour that codifies, organizes and records experiences; also, TP constructs new goals, expectations and scenarios. TP is a decisive aspect of people’s behaviour and attitudes to life [39,40], which has prevailed among the various approaches to the study of time orientations [12]. In other words, TP has acquired greater explanatory potential insofar as it has been studied in relation to specific contexts of everyday life. Five factors (also denominated perspectives), placed in the categories of past, present, and future, are derived from this perspective: the past-negative, which reflects a pessimistic, negative, or aversive attitude to the past; the past-positive, related to a nostalgic, positive construction of the past; the present-hedonistic, which is about living in the moment, immediate gratification, and pleasure-seeking; the present-fatalistic, which reflects a sense of hopelessness about the future and a limited ability to connect current behaviour to future consequences; and the future, which denotes concern with achieving goals, delaying gratification and not wasting time [40].

Each of these time factors may have a predominant or balanced presence in relation to the rest of the dimensions. If a specific orientation prevails among the factors, the individual may express a biased attitude [41,42,43,44]. On the other hand, if the orientation is balanced, the person will tend to consider the different time frames as a whole in accordance with the situational demands; in these cases, it is argued that the person is in a situation of time competence [45], with an optimal TP [39,41,46]. There have been several proposals for analysis of this idea of balance or equilibrium, which can be summed up as: having a higher score for past-positive, future, and present-hedonistic, and a lower one for past-negative and present-fatalistic TPs [42]; or displaying low scores for dysfunctional orientations (past-negative and present-fatalistic), high scores for the functional orientations (past-positive and future), and a moderate score for the present-hedonistic orientation [39,47]. In general, the form of measurement used to account for TP is the scale developed by Zimbardo and Boyd [40], which has applications in various countries [47]; in Spain, see the validation carried out by Diaz-Morales [48] and research by different authors [49,50,51,52].

As has been pointed out before, not much research has been done that relates the leisure experience with the time perspective. At a more general level, there are studies that have explored the relationship between how time is perceived and the leisure activities chosen [14,15,53]. These studies confirm that certain links between both concepts exist, including some pertaining to the dimensions of leisure. A differential contribution to this line of research is made by Cotte and Ratneshwar [54], who demonstrated that personal time perspective has a significant relationship with the benefits people pursue during their free time, which confirms time perspective as a relevant variable to leisure studies. For example, past-positive respondents were significantly more likely to seek family togetherness benefits, and present-hedonistic participants were significantly less likely than other respondents to seek spiritual benefits from recreation. More recently, research has shown [12] the impact of TP on leisure choice as well as the relationships between a balanced time perspective, available leisure time, the leisure benefits, and the chosen leisure activities. In this sense, it is necessary to explore the relationships between the time devoted to leisure and the leisure experience on the one hand, and the activities carried out and the time perspective on the other and assess gender differences in these relations.

Taking into account the corpus of prior knowledge about gender differences in the field of leisure, as well as the initial findings regarding the relationships between leisure and TP, we have derived the following three research hypotheses:

**Hypothesis** **1 (H1).** *Time dedicated to leisure activities, leisure experience, and time perspectives are related to gender, to the detriment of women compared to men*.

**Hypothesis** **2 (H2).** *A positive leisure experience (higher values of freedom perception and satisfaction) is related to positive time perspectives (past-positive, present-hedonistic, and future)*.

**Hypothesis** **3 (H3).** *A positive leisure experience (higher values of freedom perception and satisfaction) in conjunction with the positive time perspectives (past-positive, present-hedonistic, and future) and the negative ones (past-negative and present-fatalistic) is associated with specific amounts of time dedicated to leisure activities*.

The identification of the relationships and differences between time dedicated, leisure experience, and TP in men and women can reveal new characteristics that should be taken into account with respect to gender equality and inequalities in leisure.

## 2. Materials and Methods

### 2.1. Participants

A total of 869 young people (435 men and 434 women) aged between 18 and 24 years old (sample mean *M* = 21.14 years, standard deviation *SD* = 1.99) took part in the study. The original invitation to participate was made through a random-digit dialling technique, which was used to configure a convenience sample with quotas of gender and age based on the population census of Spain on 1 January 2015 [55], with a confidence level of 95% and a margin of error of 3.2%. Participation in the study was voluntary, with no payments made to the participants, who agreed to receive a unique, non-transferable link by email in order to respond online to the tools, as specified in the Procedure. This selection process followed the established, empirically tested guidelines covering the use of online panels [56,57,58].

### 2.2. Tools

There were two tools used. The first consisted of an ad hoc questionnaire with the structure and characteristics of the time budget, in line with the instrument proposed by Neulinger [32] in his research (regarding the suitability of this tool in the analysis of behaviour over time, cf. [33,36,37,38]). For this study, the recent adaptations and applications prepared for the local context were used [56,58,59]. Specifically, the questionnaire used here recorded the activities done during the day before the day on which it was answered. The register included the analytical variables of leisure developed by Codina [28]: on the one hand, the time dedicated to leisure activities during the day studied; and on the other hand, the leisure experience shaped by the perceptions of personal choice and satisfaction when doing the aforesaid activities. The incorporation of these variables, derived from Neulinger’s methodology [30,32], in the general context of the studies of use of time offers an overall view of the totality of people’s leisure activities. On the other hand, the time budget technique is used to gain a deeper knowledge of people’s leisure activities, avoiding to a large extent the social desirability that underlies specific questions (in questionnaires) about leisure activities carried out and time dedicated to them (examples of this social desirability appear in questions about physical activity and reading).

The other instrument used was the Zimbardo’s Time Perspective Inventory (ZTPI: Zimbardo and Boyd [40]), consisting of 56 items that refer to five factors: two related to the present (hedonistic, fatalistic), two to the past (positive, negative), and one to the future. This instrument was adapted to the Spanish population by Diaz-Morales [48], and has worked well with Spanish-speaking young people and adults in this and other contexts [47,49,50,51,52]. The items in the ZTPI use a Likert scale with five response options (ranging from 1 to 5, from “does not describe me at all” to “very characteristic”).

### 2.3. Procedure

The fieldwork was preceded by two preparatory phases. In the first phase, the research team worked with specialized technical staff to introduce the items into the software in the format the participants would see. To prevent data loss, the questionnaire was programmed so that, to complete it, each of the questions had to be answered in order. In other words, progress could only be made when the previous question on the screen had been answered (otherwise, the users were reminded by a pop-up window). The response categories for each question appeared on the same screen so as to avoid the need to scroll down. Once the final programming of the questionnaire had been verified, a pilot test was carried out. The results of this test were used to make any necessary adjustments to the format.

After a last performance check in the second phase of the procedure, the participants were sent an e-mail inviting them to take part in the study, with a direct link to the instrument (a single-use link, which could not be re-opened once the answers had been sent). Access to the questions was set for November 2015. The invitation was only extended to persons who met the established age requirement, being allowed to continue participating only if they gave informed consent. The ethical requirements of the Ministry of Economy and Competitiveness of Spain were applied to the current study, which means that an agreement based on the Organic Law 15/1999 on Data Protection [60] was signed—also following the recommendations from Spain’s Consejo General de Colegios de Psicólogos (General Council of Spanish Psychological Associations). According to the local and national guidelines, an additional approval for the research was not required, since data obtained with the research does not involve animals or clinical experimentation. 

The following variables were considered for a descriptive analysis of the data obtained: gender of the participants; characteristics of the leisure activities practiced (time dedicated and leisure experience, i.e., perceptions of personal choice and satisfaction); and time perspective factors (present-hedonistic, present-fatalistic, past-positive, past-negative, and future). The values for time dedicated to leisure and the leisure experience corresponded to the averages of all the leisure activities carried out during the day (from Monday to Thursday) recorded by means of the time budget. To determine whether an activity could be defined as leisure, the guidelines approved by the European Union for validating research on time use were followed [35].

The associations between the variables were calculated using the Chi Square coefficient (for gender and time dedicated to leisure activities) and Pearson’s r correlation coefficient (for perceptions of leisure experience and time perspective factors). As appropriate, gender and time dedicated were associated with the leisure experience and time perspective factors using Student’s t test or analysis of variance (statistical test based on the Fisher’s *F* ratio).

## 3. Results

Time dedication to leisure activities also presents significant differences according to gender—Table 1: χ^2^ (4, *n* = 869) = 14.50, *p* = 0.006). Women were in the majority among those who dedicate less than one hour to leisure activities (51.1% compared to 48.9% made up of men) or up to one and a half hours (58.6% women, as opposed to 41.4% men), which suggests less available leisure time in everyday life. In contrast, men predominated when the time dedicated to leisure activities was greater than two hours. Expressed in figures, between 121 and 150 min of leisure time men make up 52.2% of the total and women 47.8%, and over 150 min, 60.4% were men and 39.6% were women.

Both in the leisure experience and in the TPs, significant differences are observed according to gender (Table 2). In the case of leisure experience, women assigned higher scores than men to satisfaction: (*t*(867) = −2.07, *p* = 0.039, *d* = 0.15). This drift in the differences between men and women is also observed in the TPs corresponding to the past-positive (*t*(876) = −4.30, *p* = 0.000, *d* = 0.27), the present-hedonistic (*t*(876) = −2.43, *p* = 0.015, *d* = 0.17), and the future (*t*(876) = −2.19, *p* = 0.028, *d* = 0.16). Thus, taking the sample as a whole, gender has an influence in satisfaction related to leisure activities and the three positive TPs (past-positive, present-hedonistic, and future), with women being more positive than men.

The leisure experience in the totality of the sample studied (Table 2) presents high values both in the case of personal choice (*M* = 8.90, *SD* = 1.58) and satisfaction (*M* = 8.40, *SD* = 1.66), with the former slightly higher than the latter. In regard to TP factors, the perspectives that scored highest were the past-positive (*M* = 3.43, *SD* = 0.51), the future (*M* = 3.39, *SD* = 0.50), and the present-hedonistic (*M* = 3.33, *SD* = 0.52). These values, taking the sample as a whole, evidence the predominance of positive leisure experience and positive TP factors.

Joint analysis of the values given to leisure experience and the five TP factors (Table 3) revealed that the coefficients with greater significance (*p* < 0.001) correspond to the relationships of personal choice and satisfaction with the past-positive (respectively, *r* = 0.12 and *r* = 0.14). Other significant correlations worth highlighting are satisfaction with the present-hedonistic (*r* = 0.11) and the future (*r* = 0.09). With a *p* < 0.005, significant correlations are observed between personal choice and the present-hedonistic (*r* = 0.08), and satisfaction and the present-fatalistic (*r* = 0.08).

**Figure 1:**
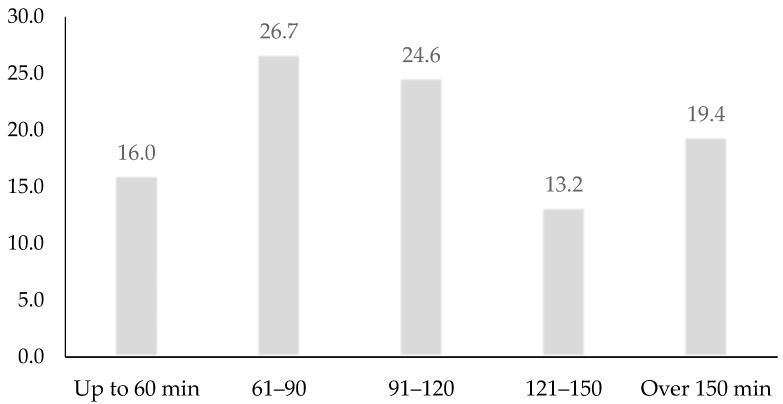
Prevalence (%) of time (minutes) spent on leisure activities.

The time dedicated to leisure activities, in 51.3% of cases, was usually between one and two hours a day, as shown in Figure 1. Specifically, a good number of participants in the study spent between 61–90 min (26.7%) or 91–120 min (24.6%) on leisure activities on weekdays. Fewer respondents spent less than one hour (16.0%) or more than two and a half hours (19.4%) on leisure activities.

The values of leisure experience and the TPs display significant differences, with a moderate effect, depending on the time dedicated to leisure activities (Table 4). In the case of leisure experience, the highest values for perception of personal choice are observed among participants who dedicated over an hour and up to two and a half hours. On the contrary, the perception of personal choice was lower when less than an hour or more than two and a half hours were devoted to leisure (statistical test based on the Fisher’s *F* ratio *F*(4, 165) = 2.62, *p* = 0.033, measure of strength of relationship in analysis of variance *ηp^2^* = 0.012). With regard to the perception of satisfaction, no significant differences were observed. In the case of the TPs, there are significant associations in three cases. Thus, the past-positive value (*F*(4, 174) = 3.16, *p* = 0.014, *ηp^2^* = 0.014) is higher among those who spent up to an hour and a half on leisure activities. On the other hand, the longer the time dedicated to leisure activities the greater the past-negative value (*F*(4, 174) = 3.38, *p* = 0.004, *ηp^2^* = 0.017)—a tendency at variance with future TP, whose values increase among those who spent less time on leisure activities.

## 4. Discussion

Testing out the hypotheses of this research has confirmed the differences between men and women regarding time dedicated to leisure activities, to leisure experience, and TPs. Furthermore, the research reveals important nuances regarding which gender scores better in each of these variables or how much leisure time is related to a leisure experience and positive TPs. Personal choice seems to suffer when the time dedicated to leisure lies at an extreme, i.e., less than an hour or more than two and a half hours. As far as the TPs are concerned, the more time devoted to leisure activities, the lower the perception of the past-positive, the greater the perception of the past-negative, and the lower the perception of the future.

### 4.1. Gendered Leisure: Pitfalls and Challenges

The differences between men and women shown in Table 1 and Table 2 confirm partially the first research hypothesis. Regarding time dedicated to leisure activities, women spend less time on a daily basis, as suggested by the hypothesis. Regarding the other two variables, differences are also found, but contradicting what was hypothesised, i.e., women enjoy better leisure experiences and more positive time perspectives than men.

In particular, in regard to the inequalities in leisure time, the significant differences identified according to gender corroborate the inequalities that negatively affect women, as shown by previous studies [3,6,8,9].

However, we have found relevant data in the leisure experience and positive TPs. Our results reveal that women rate more highly than men in both cases. In the whole set of leisure activities, the leisure experience is more positive even with less time, which suggests a need to study the reasons in greater detail. In our opinion, it would be interesting to investigate whether other variables or processes influence women’s evaluations of their leisure time, such as resilience, adaptive capacity, time management, and the fact that they are more critical when choosing their leisure activities. Also, considering that Kooij, Kanfer, Betts, and Rudolph [61] have recently seen that the future perspectives of men are more focused on work and women focus on different goals related to work, family, and leisure, we think that these differences should be valued in relation to different areas of activity of the person.

The identification of how these variables influence leisure behaviour would provide very useful knowledge for interventions with the purpose of promoting non-gendered positive personal development, proposing psychosocial interventions that satisfy both men and women. In any case, it should be highlighted that women seem to take greater advantage of the little time they have available for leisure. On the other hand, the higher scores of women in positive TPs confirm previous findings [62], specifically regarding the past-positive and the future.

### 4.2. Leisure Experience and Time Perspective: Their Positive Relations

The correlations described confirm the second hypothesis of this research, which was that a positive leisure experience, with high values for personal choice and satisfaction, correlates with positive TPs (past-positive, present-hedonistic, and future).

Regarding the scores for TP factors, these correspond to a balanced TP. The joint analysis of both variables shows that a positive leisure experience tends to correlate directly with a balanced TP, which was initially investigated by Cotte and Ratneshwar [54]. Furthermore, our findings corroborate previous findings [12], specifically expanding the explanatory potential of the relationship between TP and leisure time. To be exact, with reference to the relationships between a balanced TP and leisure choices as researched by the aforementioned authors, our research adds data which calls attention to the importance of the time dedicated to leisure activities in relation to both the leisure experience and the TP.

Regarding the relationship observed between the perception of satisfaction in leisure activities and the present-fatalistic, this finding prompts a reflection on the potential influence that negative TP factors may have on the leisure experience. In this respect, it looks as if leisure may acquire compensatory significance, serve as an escape, or signal resilience, suggesting a line of research worth deeper exploration, because as a compensatory mechanism leisure may be indicative of latent problems and, as an expression of resilience, it would account for the therapeutic potential of leisure.

### 4.3. Time Dedicated to Leisure: Benefits and Potential Risks

Time dedicated to leisure activities provides information about the boundary lying between a positive leisure experience and a negative one, which confirms, with very interesting nuances, the third hypothesis of this research. In the sample under study, it has been observed that the perception of personal choice is greater or more intense if the activity carried out lasts between an hour and two and a half hours, but lower if the activity lasts less than an hour or extends beyond two and a half hours. On the other hand, the perception of satisfaction does not vary significantly depending on the time dedicated to leisure.

The results relative to personal choice prompt a reflection on the reasons why the perception of personal choice is limited to activities with a specific duration. For example, the results showing that people who dedicate little or a lot of time to leisure make more direct use of their time. Also, these results suggest that, in the case that the least dedication is due to less available time, this limitation forces the person to make leisure choices from a more critical perspective, that is, making decisions that are more valued by himself/herself.

## 5. Conclusions

As far as future studies are concerned, they would need to go deeper into the findings of this research in order to shed new light on them. We are aware that this research has limitations, given the fact that it worked with a sample from a specific cultural context, and because a sample from one day of the week was taken as a record of activities without detailing the specific valuations of any leisure activity. However, the most important point is that the new findings suggest new lines of research.

In summary, this research shows that women devote less time to leisure than men. However, this work also shows that women, counterintuitively and despite having less time, enjoy a more positive leisure experience than men and have a more positive TP. Regarding the leisure experience and the time dedicated to it, it is clear that a positive experience of leisure occurs when the time dedicated is between one and two and a half hours—a fact that, tentatively, answers the question regarding how much leisure is necessary for people. As far as TP is concerned, it emerges that a less pessimistic view of the past is observed in those who dedicate less than an hour to leisure. This finding reveals that the amount of time dedicated to leisure activities can be a warning sign of a balanced (or unbalanced) TP.

## Figures and Tables

**Table 1:** Time spent on leisure activities. Prevalence according to gender.

	Total Sample (*N* = 869)		Men (*n* = 435)	Women (*n* = 434)	χ^2^	*p*
	*n*	%				
Time spent (min)						
Up to 60 min	139	16.0	48.9	51.1		
61–90	232	26.7	41.4	58.6		
91–120	214	24.6	50.9	49.1		
121–150	115	13.2	52.2	47.8		
Over 150 min	169	19.4	60.4	39.6	14.50	0.006

**Table 2:** Leisure experience and time perspective. Differences according to gender.

	Total Sample (*N* = 869)		Men (*n* = 435)		Women (*n* = 434)		*t*	*p*
	*M*	*SD*	*M*	*SD*	*M*	*SD*		
Leisure experience								
Freedom of choice	8.90	1.58	8.81	1.61	9.00	1.55	−1.15	0.080
Satisfaction	8.40	1.66	8.28	1.66	8.52	1.65	−2.07	0.039
Time perspective								
Past-positive	3.43	0.51	3.36	0.50	3.50	0.52	−4.30	0.000
Past-negative	3.10	0.66	3.12	0.65	3.07	0.67	1.12	0.259
Present-hedonistic	3.33	0.52	3.29	0.51	3.38	0.53	−2.43	0.015
Present-fatalistic	2.87	0.55	2.86	0.53	2.88	0.56	−0.52	0.597
Future	3.39	0.50	3.35	0.49	3.43	0.50	−2.19	0.028

Note: Leisure experience means on a scale of 0 to 10. Time perspective means on a scale of 1 to 5. *M* = sample mean; *SD* = standard deviation; *t* = statistical test based on the Student *t* distribution; *p* = probability.

**Table 3:** Intercorrelations between scores obtained for experience of leisure activities and time perspective factors.

	1	2	3	4	5	6
Leisure experience						
1. Freedom of choice	—					
2. Satisfaction	0.51 **	—				
Time perspective						
3. Past-positive	0.12 **	0.14 **	—			
4. Past-negative	0.00	−0.01	−0.01	—		
5. Present-hedonistic	0.08 *	0.11 **	0.27 **	0.15 **	—	
6. Present-fatalistic	0.02	0.08 *	0.11 **	0.39 **	0.37 **	—
7. Future	0.04	0.09 **	0.24 **	−0.03	−0.00	−0.02

Note: Leisure experience means on a scale of 0 to 10. Time perspective means on a scale of 1 to 5. * significant differences for a probability *p* < 0.05; ** significant differences for a probability *p* < 0.01.

**Table 4:** Perceived experience of leisure activities and time perspective factors according to time spent.

	Up to 60 min (*n* = 139)		61–90 (*n* = 232)		91–120 (*n* = 214)		121–150 (*n* = 115)		Over 150 min (*n* = 169)		*F*	*p*
	*M*	*SD*	*M*	*SD*	*M*	*SD*	*M*	*SD*	*M*	*SD*		
Leisure experience												
Personal choice	8.75	1.93	9.03	1.37	9.01	1.43	9.05	1.29	8.61	1.84	2.62	0.033
Satisfaction	8.35	1.86	8.38	1.62	8.55	1.42	8.42	1.57	8.26	1.87	0.77	0.543
Time perspective												
Past-positive	3.47	0.48	3.52	0.51	3.41	0.52	3.45	0.53	3.34	0.49	3.16	0.014
Past-negative	2.88	0.70	3.12	0.65	3.13	0.68	3.14	0.64	3.10	0.62	3.38	0.004
Present-hedonistic	3.32	0.55	3.39	0.51	3.29	0.52	3.34	0.53	3.31	0.50	1.15	0.329
Present-fatalistic	2.81	0.55	2.86	0.56	2.89	0.54	2.80	0.55	2.89	0.48	0.87	0.478
Future	3.47	0.50	3.41	0.52	3.38	0.48	3.37	0.51	3.31	0.48	2.26	0.061

Note: Leisure experience means on a scale of 0 to 10. Time perspective means on a scale of 1 to 5. *F* = statistical test based on the Fisher’s *F* ratio; *p* = probability.
